# Neuromuscular Performance Characteristics of Elite Colombian Sunfish Sailors: A Pilot Study

**DOI:** 10.3390/sports14050182

**Published:** 2026-05-05

**Authors:** Samuel Hormiga López, Alex Ojeda-Aravena, María Alejandra Camacho-Villa, Luis Gabriel Rangel Caballero, Jorge Enrique Buitrago-Espitia, Adrián De la Rosa

**Affiliations:** 1 Programa de Maestría en Ciencias de Deporte, Universidad Santo Tomás, Bucaramanga 681011, Colombia; samuel.hormiga@ustabuca.edu.co; 2 Departamento de Ciencias de la Actividad Física, Programa de Investigación en Deporte, Sociedad y Buen Vivir, Universidad de Los Lagos, Osorno 5290000, Chile; alex.ojeda@ulagos.cl; 3 School of Kinesiology, Universidad Bernardo O’Higgins, Santiago 8370993, Chile; 4 Pain Study Group (GED), Physical Therapy School, Universidad Industrial de Santander, Bucaramanga 680002, Colombia; 5 Performance and Health Group, Department of Physical Education and Sport, Faculty of Sports Sciences and Physical Education, University of A Coruna, 15179 A Coruña, Spain; 6 Grupo Ser, Cultura y Movimiento, Facultad de Cultura Física Deporte y Recreación, Universidad Santo Tomás, Bucaramanga 681011, Colombia; dcultu@ustabuca.edu.co; 7 Body, Physical Activity and Sport Study Group (GECAFD), Sports Department, Universidad Industrial de Santander, Bucaramanga 680002, Colombia; joebuies@uis.edu.co

**Keywords:** dinghy sailing, isometric strength, somatotype, anthropometry, rate of torque development, trunk endurance

## Abstract

**Background:** Sunfish sailing requires a combination of morphological and neuromuscular characteristics to effectively manage sail control and maintain postural stability during hiking maneuvers. **Objectives:** We aimed to describe the anthropometric and neuromuscular characteristics of elite Colombian Sunfish sailors and explore potential sex-related patterns. **Methods:** Six competitive sailors (three men and three women) underwent anthropometric assessment and somatotype calculation. Neuromuscular performance was evaluated using handgrip strength (HGS), quadriceps maximal voluntary isometric contraction, rate of force development (RFD), one-repetition maximum (1RM) lower-limb assessment, countermovement jump (CMJ), trunk endurance tests, and the Y-balance test for dynamic balance. Descriptive statistics and standardized effect sizes (Hedges’ g) were used to characterize between-sex patterns. **Results:** Anthropometric assessments suggested descriptive sex-related differences in body composition and skeletal dimensions, with a predominance of mesomorphic characteristics. Descriptive data suggested higher HGS values among men (g = 2.27–4.85), while lower-limb neuromuscular performance showed higher values among men across several RFD time windows (g = 0.81–1.45). Conversely, dynamic balance and trunk endurance outcomes showed minimal variation between sexes. **Conclusions:** This pilot study provides a preliminary physical profile of elite Colombian Sunfish sailors. The observed patterns in strength-related and morphological variables, especially HGS, quadriceps MVIC, and lower-limb power, should be interpreted as exploratory. Further research with larger samples is required to confirm these findings.

## 1. Introduction

Sailing is a multifactorial, skill-oriented endurance sport in which athletes must continuously manage interactions among wind forces, boat dynamics, and water conditions while executing precise tactical and technical actions [1,2]. These complex demands have driven substantial advances in boat design, resulting in modern dinghies that are lighter and highly sensitive to subtle variations in sailor positioning and weight distribution [3]. Consequently, physical conditioning, particularly neuromuscular performance and body composition, plays a key role in competitive sailing.

Within competitive dinghy sailing, several classes have been extensively studied due to their inclusion in Olympic and international competition programs, including the Laser, Finn, 470, and other high-performance dinghies characterized by specific demanding physical and technical requirements [1]. Research conducted on these classes has substantially contributed to our understanding of the physiological, neuromuscular, and morphological determinants of sailing performance [2,4]

Within this broader context of dinghy sailing, the Sunfish has emerged as a globally established one-design class that combines structural simplicity with considerable technical and physiological demands [3]. Competitive Sunfish regattas typically comprise several daily races on standardized buoy-marked courses, with individual race durations averaging 45 to 60 min, depending on wind conditions and course design [5]. These characteristics require sailors to integrate fine motor control, postural adaptability, and biomechanically effective strategies to sustain performance under highly variable environmental conditions [6].

Current evidence [2,7,8,9,10,11,12] indicates that competitive dinghy sailors must exhibit refined muscle control, tolerate prolonged isometric loads, respond efficiently to external perturbations, and maintain mechanically efficient postures for extended periods. These demands position the Sunfish as a valuable model for examining the neuromuscular and performance determinants of competitive dinghy sailing. However, despite the sport’s continued growth, research has focused predominantly on Olympic classes, leaving non-Olympic classes, such as the Sunfish, comparatively underexplored.

Among the physical demands faced by dinghy sailors, the hiking posture represents one of the most challenging [11]. During upwind sailing under moderate to high wind conditions, sailors project their upper bodies over the windward side of the boat to counteract the heeling moment [11,13]. This posture imposes substantial demands on the hip flexors, knee extensors, and trunk musculature, requiring sustained submaximal isometric contractions (~30–40% of maximal voluntary isometric contraction (MVIC)), interspersed with transient force peaks reported to approach or exceed MVIC [14,15]. Sustaining this posture over prolonged periods is critical for maintaining boat speed and race efficiency, underscoring the importance of local muscular endurance in dinghy sailing athletes [8,16].

Beyond sustained isometric capacity, rapid force production is also essential in dinghy sailing. The rate of force development (RFD) contributes to efficient postural corrections, rapid weight shifting, and precise maneuver execution during sudden wind fluctuations and tactical transitions [13,17]. Grip strength is a key performance-related capacity, as sailors must sustain and finely regulate high-tension loads on sheets and rigging while performing continuous adjustments throughout the race [18,19,20]. Furthermore, anthropometric characteristics, somatotype, and body composition have been shown to influence mechanical advantage, stability, and hiking tolerance across various dinghy classes, highlighting the relevance of morphological profiling in sailing performance [15,21,22,23].

Dynamic balance constitutes a key functional component of dinghy-sailing performance, as sailors must repeatedly adjust their base of support and center of mass in response to wind- and wave-induced perturbations while maintaining boat stability [20]. Functional balance tasks that challenge unilateral stance, reach capacity, and postural coordination are relevant for monitoring similar postural adjustment tasks, such as trimming and maneuver-related repositioning [24]. The integration of neuromuscular, morphological, and functional balance capacities thus appears central to performance yet remains insufficiently characterized for several non-Olympic sailing classes.

Despite these recognized demands, evidence describing the neuromuscular, anthropometric, and morphological characteristics of Sunfish sailors remains scarce. This gap is particularly evident in Colombia, where no studies have examined the physical or performance-related attributes of elite Sunfish athletes.

The lack of integrated data limits our understanding of class-specific demands and the development of evidence-based training strategies for high-performance sailors. Therefore, this study aimed to describe the neuromuscular, postural control, anthropometric, and body composition characteristics of elite Colombian Sunfish sailors and to explore potential between-sex variations in these variables.

## 2. Materials and Methods

### 2.1. Study Design and Participants

This observational and descriptive pilot study was conducted during the preparatory phase for the Star Sailor League Gold Cup Brazil 2026. Six elite sailors from the Colombian national team participated, comprising 3 females (20.33 ± 0.58 years, 158.67 ± 4.04 cm, 60.73 ± 9.17 kg, and ~175 days per sailing) and 3 males (21.67 ± 2.08 years, 174.27 ± 5.02 cm, 72.87 ± 3.17, and ~180 sailing days per year). No a priori sample size calculation was performed due to the exploratory pilot nature of the study and the limited availability of elite Sunfish sailors at the national level. Therefore, a convenience sample including all eligible athletes during the data collection period was used. Given the small sample size, the study was not designed for hypothesis testing, and all analyses were conducted with a descriptive and exploratory approach.

All sailors participated voluntarily. Participants received detailed information about all experimental procedures, and written informed consent for testing and data collection was obtained before participation. The research complied with the Helsinki Declaration, and the protocol was approved by the Ethics Committee for Human Beings at the Universidad Santo Tomás (Approval No. 06062025; 6 June 2025).

### 2.2. Selection Criteria

To be eligible, athletes had to be at least 18 years old, be currently registered with a Colombian sailing league, and regularly participate in official competitive events at national and international levels. In addition, a minimum training frequency of five sessions per week was required.

Athletes were excluded if they reported any musculoskeletal injury or pain affecting performance or training within the previous six weeks; had undergone surgery in the past year without completing the corresponding rehabilitation; had neurological or balance impairments that could influence test performance; or were unable to complete the full assessment protocol or comply with the pre-testing standardization procedures.

### 2.3. Testing Procedures

The selected tests were chosen to represent general morphological and neuromuscular capacities relevant to sailing-specific physical demands. Detailed descriptions of the anthropometric variables and neuromuscular performance assessments are presented in the following section. All assessments were conducted in the gym at the Universidad Santo Tomás on two separate days, in the morning (between 8:00 and 10:00 a.m.) and in the afternoon (between 2:00 and 5:00 p.m.). For the previous 24 h, participants were not required to perform physical exercise or training and were instructed to refrain from consuming caffeine-containing beverages, energy drinks, and pre-workout supplements.

**Figure 1:**
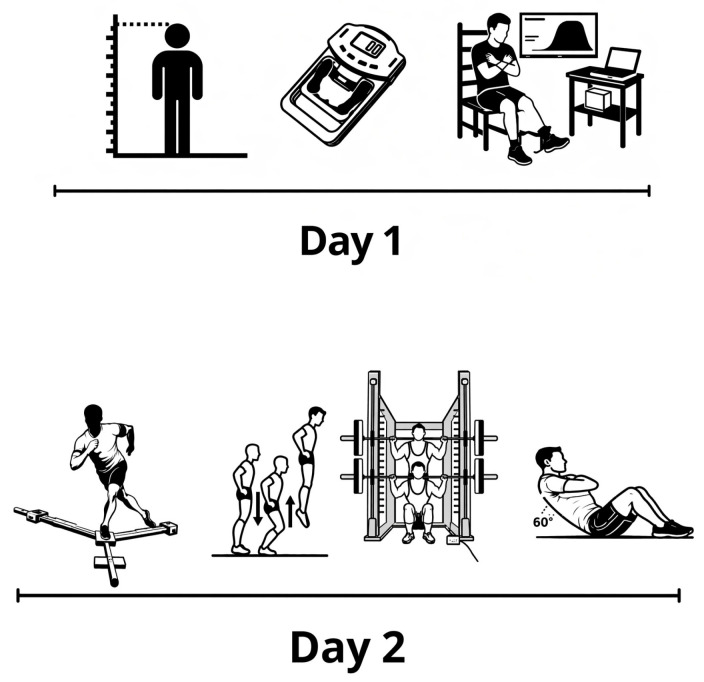
Overview of the two-session testing protocol, illustrating the sequence of anthropometric and neuromuscular assessments.

During the first gym visit, anthropometric measurements, handgrip strength (HGS), and MVIC of the knee extensors were assessed. The next day, the participants returned to the gym to perform the balance assessment, the countermovement jump (CMJ), one-repetition maximum (1RM) squat test, and core test, in that order (Figure 1). This sequence was selected to minimize potential fatigue effects by progressing from lower to higher neuromuscular demand, and standardized rest intervals were provided between tests to reduce the influence of accumulated fatigue. Dominance of the upper and lower limbs was determined via self-reported preference for throwing and kicking actions [12,13].

Anthropometric data were collected by a level 2 anthropometrist, achieving an intra-rater intraclass correlation coefficient (ICC) between 0.91 and 0.95, indicating excellent reliability.

HGS and jump performance were assessed by a second researcher trained in participant positioning and standardized verbal encouragement. Intra-rater reliability for HGS was high, with an ICC of 0.96 for the dominant hand and 0.95 for the non-dominant hand. For the CMJ test, the ICC value was 0.94, indicating very high reliability [14]. Balance, MVIC of the knee extensors, and core tests were conducted by the same researcher, who received specific training in participant positioning, device use, and software protocols.

### 2.4. Anthropometric Measurements and Body Composition

Anthropometric assessments were conducted in accordance with the standards set by the International Society for the Advancement of Kinanthropometry (ISAK). Participants wore minimal clothing and were barefoot to optimize measurement accuracy.

Height was assessed with a precision of 0.1 cm using a calibrated stadiometer (Seca^®^ 274, Hamburg, Germany; Technical Error of Measurement = 0.019%). Body mass was obtained to the nearest 0.1 kg using a digital scale (Tanita BC-240 MA, Tanita Corporation, Arlington Heights, IL, USA). Subcutaneous adiposity was evaluated through the measurement of eight skinfold sites using a calibrated skinfold caliper (Cescorf, Porto Alegre, Brazil), following standardized procedures established by the International Society for the Advancement of Kinanthropometry (ISAK). The anatomical sites included triceps, biceps, subscapular, supraspinale, iliac crest, abdominal, anterior thigh, and medial calf [25].

Girths were measured with a metal tape (Cescorf, Porto Alegre, Brazil; measurement range of up to 100 cm and accuracy to 0.1 cm) at standardized anatomical locations, including relaxed arm, flexed arm, waist, hip, and calf. Skeletal breadths were assessed bilaterally (humerus, bistyloid, and femur) using a small bone anthropometer (Cescorf, Porto Alegre, Brazil), with measurements recorded to the nearest 0.1 cm. Upper-limb segment dimensions were also evaluated on both sides, including arm length, forearm length, hand breadth, hand length, and first-to-fifth digit span. These measurements were obtained using a segmometer (Cescorf, Porto Alegre, Brazil), following previously established anthropometric protocols [26,27].

The Carter and Heath equations were used to calculate anthropometric somatotypes [28,29]:

**Endomorphy** = −0.7182 + 0.1451 × (X) − 0.00068 × (X)^2^ + 0.0000014 × (X)^3^In the equation, X corresponds to the sum of the triceps, subscapular, and supraspinale skinfolds (mm), normalized for stature through the application of the scaling factor (170.18/height in cm).Mesomorphy was derived from the anthropometric equation presented below:**Mesomorphy** = (0.858 × humerus breadth) + (0.601 × femur breadth) + (0.188 × corrected flexed arm girth) + (0.161 × corrected calf girth) − (0.131 × height (cm)) + 4.5**The ectomorphy** component was calculated using the height–weight ratio (HWR), defined as height (cm) divided by the cube root of body weight (kg). The ectomorphy classification was determined using the following criteria:If HWR was greater than or equal to 40.75, then Ectomorphy = (0.732 × HWR) − 28.58If HWR was greater than 38.25 and less than 40.75, then Ectomorphy = (0.463 × HWR) − 17.63If HWR was equal to or less than 38.25, then Ectomorphy = 0.1 [29,30]where:HWR = height/weight^1/3^The X and Y axes are used for the somatochart, calculated using the following equations: X = ectomorphy component–endomorphy componentY = 2 × mesomorphy componente − (endomorphy componente + ectomorphy componente).

Body composition was estimated according to the four-compartment model proposed by De Rose and Guimaraes (fat mass, bone mass, muscle mass, and residual mass). BF% was calculated using the Faulkner equation [31]; fat mass was calculated as BF% × body mass/100; bone mass was estimated from the bicondylar breadths of the humerus and femur [32]; residual mass was assumed as a constant proportion of total body mass [33]; and muscle mass was then determined by subtracting the combined contribution of fat, bone, and residual masses from total body mass.

In addition, the sums of three (triceps, subscapular, and iliac crest), six (triceps, subscapular, supraspinale, abdominal, anterior thigh, and medial calf), and eight skinfolds (triceps, biceps, subscapular, supraspinale, iliac crest, abdominal, anterior thigh, and medial calf) were also calculated. Furthermore, the upper arm muscle area was estimated bilaterally using the equation proposed by Frisancho [34].

All measurements were taken on the right side of the body by the same ISAK Level 2 anthropometrist to minimize interobserver variability.

### 2.5. Neuromuscular Performance Assessment

*Handgrip Strength*

Bilateral HGS was measured using a calibrated digital Hand Grip Dynamometer (Takei 5401; Tokyo, Japan) featuring a measurement range of 5–100 kg and an accuracy of 0.1 kg. The device included an adjustable handle to accommodate individual hand dimensions. Prior to data collection, sailors were provided with a standardized demonstration and verbal instructions, and the grip span was individually adjusted to ensure proper fit, following established testing procedures [26,35].

Participants stood upright in a stable bilateral stance, with the tested arm positioned alongside the body, the shoulder in adduction, and the elbow flexed at 90°. The forearm and wrist were kept in a neutral position to ensure consistent alignment during the assessment. Sailors were instructed to exert maximal force on the dynamometer for 3–5 s. Three maximal trials were performed per hand, with a 1-min rest between consecutive attempts on the same hand to minimize fatigue [36]. The highest value from each hand was recorded.

*Maximal Isometric Lower-Limb Strength*

Quadriceps MVIC was assessed at 60° of knee flexion (0° representing full knee extension) using a hand-held dynamometer (Chronojump Boscosystem, Barcelona, Spain), in accordance with a previously described protocol shown to be reliable for measuring lower-limb strength in both athletic and general populations [30,37].

**Figure 2:**
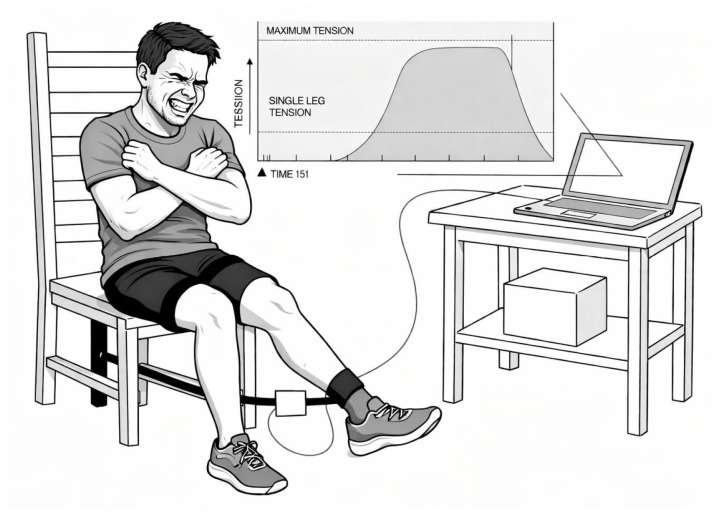
Set up of the maximal voluntary quadriceps contraction.

Participants were seated and stabilized with straps to minimize compensatory movements (Figure 2). After a standardized warm-up and two submaximal contractions, two maximal trials (~5 s) were performed per leg with 60 s rest between attempts. Strong verbal encouragement was provided. These submaximal contractions also served as familiarization trials prior to maximal efforts.

Force signals were sampled at 160 Hz and processed using the manufacturer’s software. Torque was calculated as the product of force and the moment arm (distance from the lateral femoral epicondyle to the point of force application). The trial with the highest MVIC was selected for peak torque analysis.

For RFD analyses, one trial per participant was selected based on the highest RFD value within the 0–150 ms window (RFD0–150), as this variable was considered the primary early-phase RFD metric. All RFD time windows were subsequently derived from this selected trial, to ensure consistency across variables obtained from the same contraction.

Force signals were processed using the manufacturer’s software, which automatically applies filtering to smooth the signal. Contraction onset was visually identified from the filtered force–time curve as a clear, continuous rise in torque, avoiding trials with evidence of a countermovement or non-monotonic force development [38,39]. This approach was applied consistently to all participants to improve the accuracy of RFD calculation.

*Progressive Loading Test in the Squat Exercise*

The test was conducted using free squats at the gym of the University. The athletes performed the squat on a smith machine, starting from a standing position and descending (eccentric phase) in a continuous movement until the backs of their thighs made contact with their calves. They reversed the movement and ascended back to the initial position. Participants were required to perform the concentric phase at the maximum intended velocity in all repetitions. Prior to testing, participants were familiarized with the execution technique and instructed on movement velocity requirements.

The initial load was set at 25 kg for all participants, with gradual increments of 10 kg. This progression will continue until the mean propulsive velocity (MPV) drops below 0.70 m·s^−1^ [40]. During the test, 3 repetitions were performed with light loads (MPV > 1.15 m·s^−1^), 2 with moderate loads (1.15 m·s^−1^–0.70 m·s^−1^), and only 1 with the heaviest load (MPV < 0.70 m·s^−1^).

Rest intervals were 3 min for light and moderate loads, and 5 min for heavy loads. The same progression of absolute loads was repeated for each participant. The velocity of each repetition was measured using a linear position transducer (ADR Encoder 3.0, Toledo, Spain). Considering the significant correlation (R^2^ = 0.95; SEE = 4.02%) between the percentage of one-repetition maximum (%1RM) and MPV during the full squat exercise, the 1RM was estimated from the MPV obtained with the heaviest load used in the test, according to the following equation [40]:

%1RM = −5.961 · MPV^2^ − 50.71 · MPV + 117.0

This equation has been used in different populations performing the full squat exercise. In the present study, it was applied to obtain a standardized estimate of relative strength under controlled testing conditions,

*Vertical jump test*

Before testing, sailors completed a standardized warm-up that included light jogging, skipping, half-squats, lunges, and leg swings. Then, participants familiarized themselves with the testing protocol before performing three maximal effort countermovement jumps (CMJs) on a dual-force plate system (Delta Force Plate; Kinvent; V2; 2000 Hz; Montpellier, France), which was recalibrated for each participant. Data were recorded using the Kinvent Physio App (v2.7.1). To enable comparisons between groups, mean forces were normalized to body mass (N/kg).

Each trial started from an upright standing position, with feet placed hip-width to shoulder-width apart and hands on the hips to eliminate arm swing. Participants were instructed to distribute their weight evenly across both force plates before beginning the movement. From this position, they performed a quick downward movement to approximately 90° of knee flexion, then immediately executed a maximum vertical jump and landed in a controlled, athletic stance on the force plates [41].

After each attempt, participants returned to the starting position before starting the next jump. A total of three valid trials were required; jumps were repeated if participants removed their hands from their hips or showed excessive knee flexion during flight. To reduce fatigue, a 60-s rest period was provided between trials, and the average of the three valid jumps was used for later analysis [42,43]. During testing, participants received verbal encouragement to promote maximum effort and force production. Force-time variables across the eccentric, isometric, and concentric phases of the movement were included in the analysis.

*Trunk muscle endurance tests*

The McGill trunk muscular endurance test was used to assess the resistance of the trunk flexors, lateral flexors, and extensors by measuring how long the isometric positions can be held (holding time). These are the most reliable core endurance tests and are frequently used in clinical settings to evaluate the isometric endurance of core muscles [44].

*Trunk flexor endurance test*

**Figure 3:**
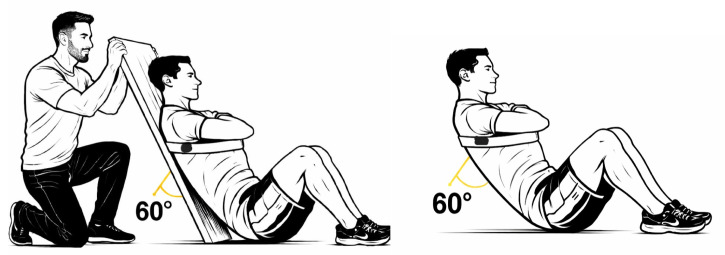
Trunk flexor endurance test.

The sailors were asked to adopt a starting position sitting with their hips and knees bent at 90°, crossing their hands over their chest, and touching their shoulders. Then, they were instructed to incline the trunk to 60° and sustain the position following the removal of the board [44]. Verification of knee and trunk flexion was performed using an electronic goniometer (K-Force Sensor, Kinvent, Montpellier, France). The goal was to sustain this posture without back support for as long as possible, and the test concluded when the participant’s initial position was altered (Figure 3). A digital timer was used to record the duration from the moment the board was removed until the test ended. The results were registered in a Microsoft Excel sheet.

*Trunk lateral flexor endurance test*

**Figure 4:**
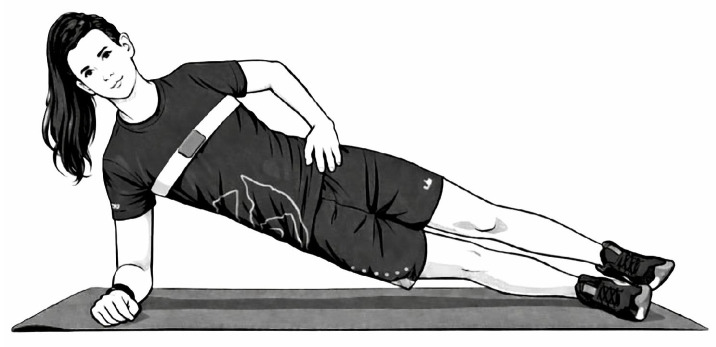
Trunk lateral flexor endurance test.

Athletes adopted a lateral decubitus position, supported on the forearm with the elbow flexed at 90°. Legs were extended while maintaining alignment of the body segments, forming a straight line from the shoulders to the hips and feet. The foot of the non-preferred leg was positioned in front of the preferred leg, while the free arm rested on the contralateral shoulder (Figure 4). Participants were instructed to maintain this position for as long as possible until volitional exhaustion, while the examiner provided continuous feedback to ensure correct posture [45,46]. A digital timer (HS-70W-1DF, Casio Electronics Co., Ltd., Guangzhou, China) was initiated when participants lifted their hips off the floor and stopped when the position was not sustained for more than 3 s. The test was repeated on the opposite side using the same procedure [44].

*Trunk extensor endurance test*

**Figure 5:**
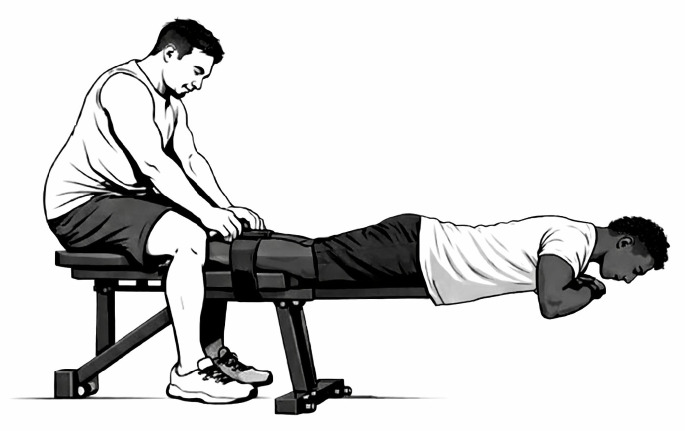
Trunk Extensor Endurance Test.

Sailors sustained a horizontal and prone posture on a treatment table, with their anterior superior iliac spines aligned with the table edge. The upper body was supported on a chair using the arms, and the pelvis and lower limbs were secured to the table with three stabilization straps. Upon readiness, participants lifted their torso until it was parallel to the floor and aligned with the lower limbs, with their arms crossed over their chest (Figure 5). Timing began once this position was assumed and continued until the participants could no longer sustain the posture, at which point the time was recorded.

### 2.6. Dynamic Balance Assessment

The lower-quarter YBT (YBT-LQ) is a functional assessment commonly used to quantify dynamic postural control in the anterior, posterolateral, and posteromedial directions. This test has demonstrated good inter-rater reliability, with a standard error of measurement reported at 3.1–4.2 cm [47].

Dynamic balance was assessed using the YBT-LQ with a commercially available device (Y Balance Test, Move2Perform, Evansville, IN, USA). Before testing, limb length on the right side was measured from the anterior superior iliac spine to the distal tip of the medial malleolus and recorded to the nearest 0.5 cm using a metal tape (Cescorf, Porto Alegre, Brazil; range up to 100 cm; resolution 0.1 cm). Participants received standardized verbal instructions accompanied by a live demonstration.

Following familiarization (three practice trials per direction), testing was performed bilaterally in the anterior (ANT), posteromedial (PM), and posterolateral (PL) directions, with three valid attempts recorded for each condition. Trials were considered invalid if participants failed to maintain proper test execution, including contact with the push box, inability to return to the starting position in a controlled manner, use of the reach indicator for support, loss of single-leg stance, unintended ground contact, or placement of the reaching foot on top of the push box [48]. Reach distances were recorded to the nearest centimeter, and the highest value obtained in each direction was retained for subsequent analysis.

Inter-limb asymmetry was examined by comparing the maximal reach distances obtained with the dominant and non-dominant legs in each direction. Reach performance was normalized to limb length by expressing each directional distance as a percentage of leg length (reach distance/limb length × 100). A composite score (COMP) was subsequently calculated as the mean of the three normalized directional scores [49].

$$\text{Composite score}\;(\mathrm{COMP})=\frac{(\text{sum of the three reach distances})\times 100}{3\times \text{limb length}}$$

### 2.7. Statistical Analysis

All analyses were performed in R (R Foundation for Statistical Computing, Vienna, Austria). Data are presented as mean ± standard deviation. Given the exploratory design and very small sample size (n = 3 per group), analyses were primarily descriptive.

Between-sex variation was quantified using Hedges’ g (men − women), with 95% confidence intervals (CI) estimated via bias-corrected bootstrap resampling (5000 iterations). Bootstrap resampling was used to characterize the uncertainty around the estimates without relying on distributional assumptions, and the resulting intervals reflect the expected variability associated with the small sample size.

Effect sizes were calculated to describe the direction and relative magnitude of between-sex variation, and no categorical interpretation based on conventional thresholds was applied.

## 3. Results

### 3.1. Anthropometric and Body Composition Profile

Men presented higher mean values for height and body mass compared to women (Table 1), with standardized differences observed for height (g = 2.73) and body mass (g = 1.45). Sitting height showed a standardized difference (g = 0.92), suggesting a similar proportional trunk length between sexes despite overall stature differences.

A consistent pattern was observed across skinfold measurements, with women showing higher values at most individual sites. Negative standardized differences were observed for triceps (g = −3.20), medial calf (g = −3.97), and front thigh (g = −2.79) skinfolds. When aggregated, the sum of 6 and 8 skinfolds also showed negative standardized differences (g = −2.38 and g = −1.72, respectively).

In contrast, girth and breadth measurements tended to show higher values in men. Upper-limb circumferences, particularly arm flexed and tensed (g = 1.19), and skeletal breadths including humerus (g = 1.97), bistyloid (g = 2.07), and femur breadth (g = 2.07), showed positive standardized differences (Table 1).

The upper-limb muscle area showed positive standardized differences for both the dominant (g = 2.29) and non-dominant sides (g = 2.23) (Table 2). Regarding body composition, bone mass (g = 2.96) and residual mass (g = 2.67) showed positive standardized differences in men, whereas absolute fat mass showed values close to zero (g = −0.19). Muscle mass showed a positive standardized difference (g = 1.08), indicating higher lean mass in men.

### 3.2. Somatotype Characteristics

Somatotype analysis indicated a mesomorphic predominance in both sexes. Higher values were observed for endomorphy in women (g = −1.12) and for ectomorphy in men (g = 1.21), whereas mesomorphy showed values close to zero (g = 0.30) (Table 3).

**Figure 6:**
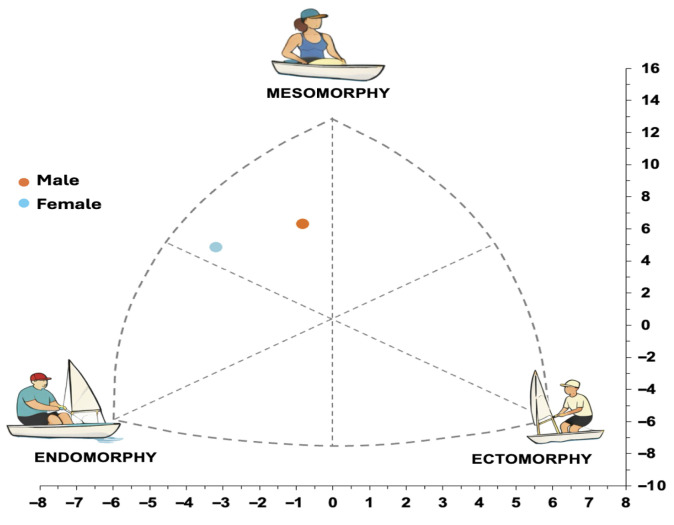
Sex-specific somatotype distribution in Elite Sunfish Sailors. Mean Somatotypes of men (n = 3) and women (n = 3) Elite Sunfish Sailors plotted according to the Health–Carter anthropometric method. Somatochart coordinates were derived as X = ectomorphy–endomorphy and Y = 2 × mesomorphy − (endomorphy + ectomorphy). Each symbol represents the sex-specific group mean.

As illustrated in the somatochart (Figure 6), both groups were positioned within the mesomorphic region, with a relative shift toward the endomorphic component in women and toward the ectomorphic component in men

### 3.3. Neuromuscular Performance Test

**Figure 7:**
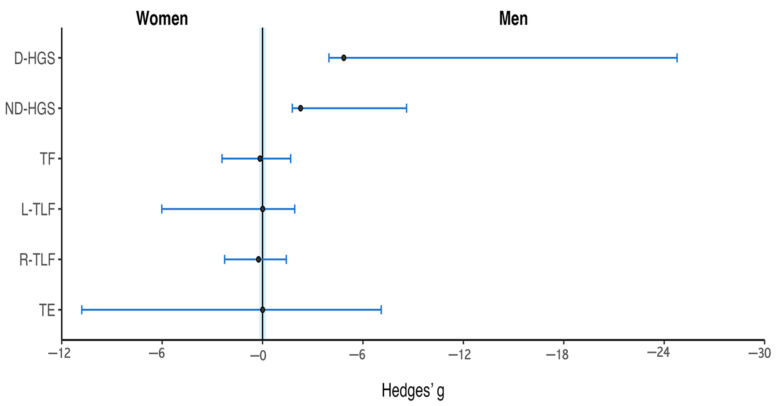
Exploratory standardized differences in upper-limb and trunk neuromuscular performance tests between sexes. Hedges’ g values with 95% confidence intervals are presented for dominant handgrip strength (D-HGS), non-dominant handgrip strength (ND-HGS), trunk flexion (TF), left trunk lateral flexion (L-TLF), right trunk lateral flexion (R-TLF), and trunk extension (TE) in Elite Sunfish Sailors (n = 3 men; n = 3 women). Effect sizes were calculated as men − women; positive values indicate higher scores among men, whereas negative values indicate higher scores among women. The vertical reference line at g = 0 indicates there are no differences between sexes.

Handgrip strength showed higher values in men on both the dominant (g = 4.85) and non-dominant sides (g = 2.27) (Supplementary Table S1), whereas trunk isometric endurance tests showed values close to zero (Figure 7).

**Figure 8:**
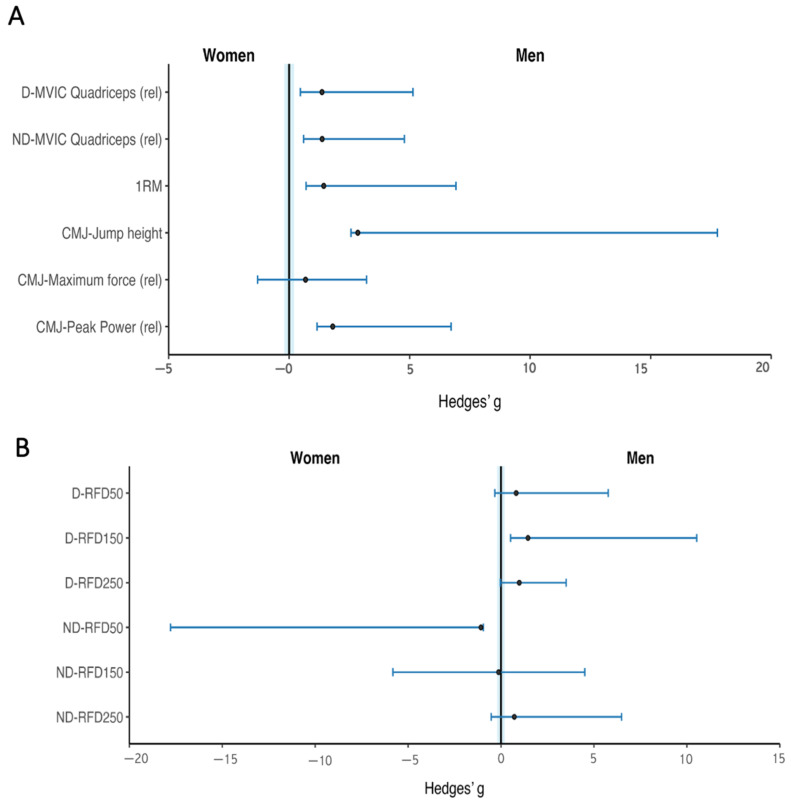
Exploratory standardized differences in lower-limb neuromuscular performance tests between sexes. (**A**) Maximal voluntary isometric force (MVIC), Countermovement jump height (CMJ), and one-repetition maximum (1RM). (**B**) Rate of force development (RFD). Hedges’ g values with 95% confidence intervals for lower-limb neuromuscular performance in elite Sunfish sailors (n = 3 men; n = 3 women). Effect sizes were calculated as men − women; positive values indicate higher scores among men, whereas negative values indicate higher scores among women. The vertical reference line at g = 0 indicates there are no differences between sexes.

Men showed higher MVIC values for the quadriceps on both the dominant (g = 1.36) and non-dominant sides (g = 1.37), as well as for 1RM (g = 1.43). CMJ height (g = 2.85), CMJ peak power (g = 1.80), and CMJ maximum force also showed a positive standardized difference (g = 0.68) (Figure 8A).

Standardized mean differences for RFD across time intervals are shown in Figure 8B. In the dominant side, positive standardized differences were observed at 0–150 ms (g = 1.45), 0–50 ms (g = 0.81), and 0–250 ms (g = 0.98) (Supplementary Table S1).

In the non-dominant side, RFD at 0–50 ms (g = 1.08) and 0–250 ms (g = 0.71) demonstrated positive standardized differences, whereas RFD at 0–150 ms showed values close to zero (g = −0.13) (Figure 8B).

### 3.4. Dynamic Balance

Standardized differences in dynamic balance were generally close to zero for the anterior, posteromedial, and composite directions for both the dominant and non-dominant limbs (Table 4). A higher value was observed in the non-dominant posterolateral direction (g = 2.52), whereas the remaining variables showed minimal variation.

## 4. Discussion

The present pilot study aimed to provide a characterization of elite Colombian Sunfish sailors within the multifactorial framework of dinghy-sailing performance. The selected assessments were intended to reflect key physical components relevant to sailing tasks, including sustained isometric force during hiking, postural control under unstable conditions, and rapid force production in response to wind variability. Consistent with this context, descriptive differences were observed in anthropometric and neuromuscular strength variables, whereas core endurance and balance outcomes were more comparable between sexes.

Given the small sample size and the width of the confidence intervals for several variables, these findings should be interpreted as indicative of general patterns rather than inferential evidence. Accordingly, the results are best understood within an exploratory and descriptive framework.

### 4.1. Anthropometric, Somatotype, and Body Composition

Anthropometric references for dinghy sailors across different classes have consistently shown a profile more closely associated with relatively low skinfold thicknesses and greater skeletal breadths and limb lengths, characteristics that may contribute to mechanical leverage and stability during hiking maneuvers. The findings of the present study align with previous reports describing similar anthropometric patterns in sailors competing in different dinghy classes [21,22,23].

There is evidence showing that the mesomorphic component is dominant among sailors across various sailing classes in which somatotype has been evaluated, including Finn [23,50], Formula [51], RSX [50], Optimist [52], and Olympic classes such as 470 and 49er [50]. Although, to our knowledge, no research has reported morphological, somatotype, or body composition characteristics in sunfish sailors, the results of the athletes in the present study are comparable to those of Olympic classes [23,53]. For example, research by Pezelj among elite male sailors in the ILCA 7 and Finn classes reported values for several skinfold measures that were either similar to or higher than those reported in our study. They also reported a lower mesomorphy component among ILCA 7 sailors than in our study, suggesting relatively greater musculoskeletal development in our athletes.

It can be hypothesized that, similar to other sailing classes, the Sunfish class may be associated with a dominant mesomorphic component, which could relate to the class’s specific mechanical demands and be key to maintaining prolonged hiking positions and generating continuous isometric force to keep the boat stable.

On the other hand, the present study observed descriptive sex-related differences in height, body weight, limb length, and skeletal breadths, with higher values observed in males (Table 1). These variables are biomechanically relevant in the context of hiking, where righting moment is generated by projecting body mass windward [11,24].

Evidence from Olympic sailing classes indicates that body mass and quadriceps strength are associated with hiking performance and fatigue resistance [15,21,22]. In line with this, Pan & Sun [8] reported relationships between physical characteristics and performance outcomes, whereas Pezelj et al. [23] described distinct somatotype profiles in elite Finn sailors. Together, these findings suggest that morphological characteristics may be relevant for mechanical aspects of dinghy sailing, as reported in other sailing populations [8,15].

Conversely, in the present study, somatotype findings for the mesomorphy component were relatively similar between sexes (Table 3), with women showing relatively greater endomorphy and men showing greater ectomorphy. According to Heath–Carter, ectomorphy reflects relative linearity, while endomorphy represents relative adiposity [28].

This pattern may be partly related to descriptive sex-related differences, in which greater body linearity and skeletal breadth could potentially contribute to mechanical leverage hiking, whereas a higher endomorphic component observed in women may reflect differences in body composition distribution rather than functional limitation.

Although hiking performance was not directly assessed, the sustained isometric demands of the task suggest that morphological characteristics may be relevant for mechanical leverage during sailing maneuvers.

### 4.2. Handgrip Strength

Upper-limb force production is considered a key neuromuscular capacity involved in sheet handling during dinghy sailing [19]. During a race, sailors must continuously control sheet tension and make frequent trimming adjustments while maintaining balance and counteracting forces that cause the boat to heel [54]. These tasks demand sustained activation of the forearm flexor muscles and precise regulation of grip force to control the rope under load throughout sailing maneuvers [55]. Previous studies in sailing environments have demonstrated that extended rope handling and sail adjustments can cause significant neuromuscular fatigue and physiological stress [19,56]. Therefore, maintaining adequate HGS levels may help sailors effectively control the sails and delay fatigue during lengthy sailing tasks.

In the present study, HGS showed large descriptive standardized differences between the sexes, with higher values in men. These patterns appear consistent with the morphological characteristics observed in our sample, including greater upper-arm muscle area, skeletal breadths, and overall muscle mass in men (Table 1 and Table 2).

Similar relationships between upper-limb morphology and HGS have been reported in other athletic populations [27,57], including rowing and overhead sports, where greater muscle mass and skeletal dimensions are associated with higher grip force production capacity [26,56]. Although direct comparisons with sailing performance should be made cautiously, these findings may indicate that upper-limb structural characteristics may be associated with the capacity to generate grip force in athletes exposed to repeated load-bearing tasks involving the hands and forearms [55].

Results in the present study are lower than those reported in male and female elite rowers [19,56] and professional offshore sailors. This difference may be partly explained by the lower absolute mechanical demands associated with sheet handling in the Sunfish class compared to rowing and larger sailing vessels. In addition, the intermittent nature of trimming actions and the greater reliance on whole-body coordination and hiking-related stabilization may reduce reliance on maximal grip force capacity in this population.

### 4.3. Neuromuscular Strength and Power Characteristics

One of the most important techniques during competition is hiking, in which the sailor extends the body outward over the windward side of the boat while keeping the feet secured under hiking straps, thereby increasing the righting moment and reducing excessive heeling [58]. During hiking, sailors perform sustained isometric contractions of the quadriceps and trunk muscles in response to wind and wave condition [59]. Consequently, both maximal force production and the ability to rapidly develop force are considered key neuromuscular factors for sailing performance [60,61]

Consistent with this, Niinimaa et al. [54] highlighted the important role of quadriceps strength in sailing performance. The authors reported that the average quadriceps MVIC in sailors (1045 N) exceeded that in swimmers (720 N) and oarsmen (741 N), emphasizing the high strength demands of dinghy sailing. Additionally, quadriceps strength has been strongly linked to the hiking moment at full knee extension (HM_180_) [62], a biomechanical measure of the sailor’s ability to generate righting torque while maintaining the hiking stance.

Quadriceps torque has been evaluated in multiple studies using different knee and trunk angles during measurement, which makes direct comparisons with our results challenging. For example, Aagaard et al. [62], using knee and hip–trunk angles of 45° and 100°, respectively, reported higher MVIC values (323 Nm and 228 Nm for male and female elite sailors) compared to those observed in this study.

In particular, variations in hip–trunk angle can influence the effective moment arm and, consequently, torque production during testing. More extended trunk positions may increase the moment arm relative to the knee joint, potentially allowing greater torque values to be produced.

This methodological variability may also account for differences observed when comparing our findings with those reported in other sailing populations. Similarly, the female values in our study were slightly lower than those reported by Friesenbichler et al. [63] in a sample of 470er and Laser female class sailors. In this study, the hip–trunk angle was set at 90°, whereas Friesenbichler et al. used 135°, increasing the effective lever arm of the upper body relative to the knee joint and potentially enabling greater torque production during the test.

The results for lower-limb strength and power in this study differ from those reported in previous studies [1,19,21,64]. For example, male sailors in this study showed higher 1RM values compared to those in 470 Helmsmen and 470 Crew classes. However, female Sunfish sailors in our study achieved lower but comparable results on this variable compared to those reported in the Laser Radial and 470 Crew classes, but higher than in the 470 Helmsmen class [1]. Additionally, regarding CMJ variables, our female Sunfish sailors are compared with recent reports by Pan et al. [21] across different sailor classes.

Collectively, our findings suggest that the sailors included in the present study exhibit physical characteristics broadly comparable to those reported in athletes from other sailing classes [20]. Although some trends were observed across specific strength and power variables, the overall profile of lower-limb neuromuscular performance appears to fall within the range previously reported for competitive dinghy sailors.

On the other hand, this study identified possible sex-based descriptive differences in all lower-limb neuromuscular performance variables studied, with males showing higher values and larger effect sizes than females (Figure 8A and Supplementary Table S1). These differences are consistent with well-established physiological distinctions between sexes. Males generally present greater skeletal muscle mass and cross-sectional area, particularly in the lower limbs, which may be related to differences in force and power production capacity. Although segmental lower-limb muscle mass was not estimated in our study, a large effect size (g = 1.08) was observed for overall muscle mass (Table 2).

This greater muscle mass has been previously associated with higher circulating testosterone levels, which promote muscle protein synthesis, strength, and hypertrophic adaptations [65]. In addition, males typically exhibit a higher proportion of type II muscle fibers and greater absolute neural drive during maximal contractions, factors that further enhance the ability to generate force rapidly [66,67]. However, these mechanisms were not directly assessed in the present study.

During the hiking maneuver, rapid force production is especially important in variable wind conditions, where sailors must repeatedly shift body mass in response to gust-induced heeling moments [7,14,15]. In this study, RFD was measured in three-time windows (0–50 ms, 0–150 ms, and 0–250 ms) during the MVIC of the quadriceps to assess the early-phase neuromuscular capacity of the knee extensors to generate force quickly.

Regarding RFD values, males showed relatively higher RFDs than females in most cases, suggesting a potential difference in rapid force during the early stages of contraction. Interestingly, females exhibited higher RFD values in the 0–50 ms window than males on the non-dominant side. These results may be partially related to the morphological characteristics of the sample (Table 1). Since males are heavier and taller than females, they may have a greater potential to generate counterbalance when the wind hits the sail, helping to keep the dinghy under control more effectively than females. In this context, it is possible that different neuromuscular strategies may be involved during the hiking maneuver under variable wind conditions, particularly in the early-phase RFD (0–50 ms window). In this regard, early-phase RFD (≤50 ms) is known to depend predominantly on neural mechanisms, particularly the rapid recruitment of motor units and the initial motor neuron discharge rate, rather than on muscle cross-sectional area [68]. Because females typically have lower muscle mass and lower maximal strength, they may rely proportionally more on rapid neural activation strategies to generate force quickly at the onset of contraction [69].

Additionally, the asymmetric tactical demands of Sunfish racing may contribute to this pattern. During competition, sailors frequently sail on starboard tack due to right-of-way rules, particularly during the start and when rounding the windward mark.

This repeated sailing orientation may create asymmetric stabilization demands, requiring rapid force production to counteract sudden increases in heeling moment. However, this interpretation is hypothetical and was not directly assessed in the present study. Under these conditions, the non-dominant limb may contribute more to rapid stabilization of the sailor–boat system, which could partly explain the higher early-phase RFD observed in females on that side in the present sample.

### 4.4. Trunk Endurance

The trunk endurance plays an important role in maintaining stability during the hiking maneuver [62]. This posture requires coordinated activation of the trunk and lower limbs to maintain spinal alignment and ensure effective force transmission between body segments, while counteracting heel-heel forces [8,15,16]. Trunk endurance outcomes showed minimal variation between sexes in the present study (Figure 7, Supplementary Table S1).

Although the McGill tests cannot replicate the dynamic conditions experienced during sailing, they provide an objective measure of sustained trunk muscular endurance. Spurway [9] and Bourgois et al. [15] highlighted the role of trunk musculature in supporting prolonged quasi-isometric efforts during hiking [20]. Thus, the observed similarity among participants may suggest that trunk endurance could represent a relevant physical capacity among elite Sunfish sailors [70]. When compared with the results in the Helmsmen and Crew classes reported by [21], the Sunfish Sailors in the present study performed better on the trunk flexion test in both males and females. However, reports in Optimist sailors were similar to ours [71]. These findings may reflect class-specific physical demands, as both Sunfish and Optimist sailing require sustained trunk stabilization to maintain balance and boat control during prolonged hiking or counterbalancing postures. In contrast, classes involving helmsman–crew interaction may present more dynamic movement patterns, potentially reducing the continuous loading of the trunk flexor musculature. This could partly explain the higher trunk flexion endurance observed in the present sample.

However, modern perspectives on trunk function emphasize the importance of dynamic neuromuscular coordination and stabilization rather than static endurance alone [62]. Therefore, future research should incorporate electromyographic and biomechanical analyses of sailing-specific trunk muscle activation to better understand trunk muscle activation patterns during on-water performance under wind variability.

### 4.5. Functional Dynamic Balance

Sailing is a dynamic sport characterized by highly variable environmental conditions, including wind, water state, and interactions with other sailors to maintain stability and optimal trim [72]. Sailors perform maneuvers such as hiking and tacking, both of which demand effective dynamic balance and postural control to counteract external forces [12,73].

Despite this, the assessment of balance in sailing remains limited because current methods do not fully capture the mechanical and environmental challenges of the sport. Among the most common and reliable methods for assessing dynamic balance, the YBT measures functional reach distance normalized to limb length [48]. Although the test does not mimic the external disturbances typical of sailing, it offers a standardized measure of dynamic stability during self-induced movements in various directions [73].

In the present study, a possible large effect size was observed only on the non-dominant side, favoring females (Table 4). It is noteworthy that females also exhibited a higher effect size in the early-phase RFD on the same side than males (Figure 8A and Supplementary Table S1). It has been described that rapid force production during the early phase of contraction may contribute to postural stabilization, as early RFD is strongly influenced by neural activation and is critical for generating corrective joint torques in response to sudden perturbations [39]. Building on that point, it is reasonable to think that during sailing, where wind gusts can rapidly alter the heeling moment of the boat, the ability of females to produce force rapidly in the non-dominant side may contribute to maintaining their stability during tack maneuvers.

Finally, although no previous findings in Sunfish sailors have been reported, our results are comparable to those reported by Beyza et al. [68] in a sample of Windsurfing, Optimist, and Laser sailors.

### 4.6. Pilot Nature and Future Directions

This study represents the first integrated profiling of elite Colombian Sunfish sailors. The limited sample size restricts inferential conclusions; thus, findings should be interpreted descriptively. Additionally, the confidence intervals observed reflect the level of uncertainty expected with a very small sample and should be interpreted as indicative of variability around the observed patterns rather than precise estimates of effect magnitude. It should also be noted that no direct on-water performance or sailing-specific outcome measures were included. Therefore, the relationships between the assessed variables and actual sailing performance remain indirect and should be interpreted within the exploratory scope of the study

Future research should combine morphological profiling, neuromuscular testing, hiking force measurement, Global Navigation Satellite System (GNSS) race analysis, and longitudinal performance data to clarify class-specific determinants in Sunfish sailing.

## 5. Conclusions

This pilot study provides the first characterization of the anthropometric, somatotype, and neuromuscular profiles of elite Colombian Sunfish sailors. A predominance of mesomorphic characteristics was observed in both sexes, with descriptive differences in strength-related and morphological variables, particularly in HGS, quadriceps MVIC, and lower-limb power.

In contrast, trunk endurance and dynamic balance showed minimal variation between sexes, suggesting that these abilities may be relevant for maintaining postural stability during sailing maneuvers. These results should be viewed as a descriptive, preliminary, exploratory framework, given the study’s design features.

Future research with larger samples and sailing-specific biomechanical assessments is necessary to confirm these findings and deepen our understanding of the morphological and neuromuscular traits associated with the physical demands of the Sunfish class.

## Figures and Tables

**Table 1:** Anthropometric profile of male and female Elite Sunfish Sailors (n = 6).

Variables	Male (Mean ± SD)	Female (Mean ± SD)	g [95% CI]
Basic			
Height (m)	1.74 ± 0.05	1.58 ± 0.04	2.73 [2.19–7.74]
Sitting height (cm)	91.62 ± 4.10	86.93 ± 3.94	0.92 [−0.27–3.88]
Weight (kg)	72.87 ± 3.17	60.73 ± 9.16	1.45 [0.86–7.96]
Skinfolds (mm)			
Triceps	7.83 ± 2.02	16.51 ± 2.30	−3.20 [−13.71–−2.41]
Subscapular	9.67 ± 1.53	14.52 ± 6.54	−0.81 [−8.49–0.18]
Biceps	4.33 ± 1.04	6.01 ± 2.51	−0.69 [−4.55–0.90]
Abdominal	12.16 ± 3.62	15.83 ± 5.03	−0.67 [−4.81–0.54]
Iliocrestal	10.67 ± 3.75	12.67 ± 6.65	−0.29 [−2.74–1.67]
Supraspinal	5.83 ± 1.61	7.67 ± 4.19	−0.46 [−4.89–5.22]
Front thigh	8.67 ± 2.56	18.51 ± 3.04	−2.79 [−17.63–1.90]
Medial calf	6.51 ± 1.32	15.17 ± 2.08	−3.97 [−24.17–−3.54]
∑ 3 skinfolds	28.17 ± 7.25	43.67 ± 14.63	−1.07 [−6.01–−0.45]
∑ 6 skinfolds	50.67 ± 8.61	88.17 ± 15.61	−2.38 [−11.54–−2.04]
∑ 8 skinfolds	65.67 ± 12.29	106.83 ± 24.15	−1.72 [−22.21–−1.42]
Girths (cm)			
Arm relaxed	29.33 ± 2.08	27.67 ± 1.52	0.73 [−0.413–4.24]
Arm flexed and tensed	32.67 ± 1.53	30.01 ± 2.03	1.19 [0.23–4.58]
Waist	76.33 ± 0.58	71.33 ± 10.11	0.56 [−12.41–22.21]
Hip	96.02 ± 1.04	99.67 ± 6.65	−0.61 [−15.03–4.52]
Thigh medial	54.01 ± 2.65	51.02 ± 3.04	0.84 [−0.214, 10.45]
Calf	36.67 ± 0.57	34.33 ± 2.51	1.02 [−0.65–8.49]
Bone breadth (cm)			
Humerus	7.07 ± 0.40	6.07 ± 0.41	1.97 [1.29–6.40]
Bystyloid	5.87 ± 0.47	5.01 ± 0.01	2.07 [1.74–6.21]
Femur	10.04 ± 0.25	9.33 ± 0.28	2.07 [1.75–4.64]
Lengths (cm)			
Arm	33.33 ± 1.53	31.01 ± 1.01	1.45 [0.82–5.22]
Forearm	27.67 ± 0.58	24.51 ± 1.32	2.48 [1.94–11.10]
Hand	19.83 ± 0.76	18.01 ± 0.87	1.79 [1.31–7.19]
First-to-fifth finger distance	21.83 ± 0.58	20.01 ± 1.03	1.79 [1.14–5.55]
Arm (left)	33.33 ± 1.53	31.01 ± 1.04	1.45 [0.83–4.58]
Forearm (left)	27.33 ± 0.58	24.17 ± 1.26	2.58 [1.83–8.50]
Hand (left)	19.83 ± 0.76	18.01 ± 0.87	1.79 [1.30–7.18]
First-to-fifth finger distance (left)	22.34 ± 1.04	20.01 ± 1.02	1.83 [1.27–6.53]

CI: confidence interval. Values are presented as mean ± standard deviation. Hedges’ g represents the bias-corrected standardized mean difference between sexes. Hedges’ g was calculated as Male–Female. Positive values indicate higher scores among males. Given the exploratory design and small sample size (n = 3 per group), emphasis is placed on effect magnitude and direction rather than statistical inference.

**Table 2:** Body composition profile of Elite Sunfish Sailors (n = 6).

Variables	Male (Mean ± SD)	Female (Mean ± SD)	g [95% CI]
Faulkner body fat (%)	11.21 ± 1.29	14.12 ± 2.55	−1.15 [−5.16–−0.82]
Upper arm muscle area—DS (cm^2^)	57.65 ± 8.10	40.26 ± 2.87	2.29 [1.54–33.24]
Upper arm muscle area—NDS (cm^2^)	56.23 ± 8.01	40.13 ± 1.62	2.23 [1.45–17.12]
Fat mass (kg)	8.18 ± 1.06	8.69 ± 2.73	−0.19 [−3.86–3.61]
Bone mass (kg)	12.27 ± 1.09	9.08 ± 0.54	2.96 [2.03–30.57]
Residual mass (kg)	17.56 ± 0.76	12.69 ± 1.92	2.67 [2.03–30.57]
Muscle mass (kg)	34.86 ± 1.74	30.28 ± 4.44	1.08 [0.71–6.93]

DS: dominant side; NDS: non-dominant side; CI: confidence interval. Values are presented as mean ± standard deviation. Hedges’ g represents the bias-corrected standardized mean difference between sexes with 95% confidence intervals. Hedges’ g was calculated as Male–Female. Positive values indicate higher scores among males. Given the exploratory design and small sample size (n = 3 per group), emphasis is placed on effect magnitude and direction rather than statistical inference.

**Table 3:** Somatotype characteristics of elite Sunfish sailors (n = 6).

Variables	Male (Mean ± SD)	Female (Mean ± SD)	g [95% CI]
Endomorphy	2.78 ± 0.80	4.30 ± 1.32	−1.12 [−6.22–−0.46]
Mesomorphy	5.53 ± 1.21	5.14 ± 0.79	0.30 [−1.46–11.27]
Ectomorphy	1.95 ± 0.57	1.11 ± 0.54	1.21 [0.28–57.40]

CI: confidence interval. Values are presented as mean ± standard deviation. Hedges’ g represents the bias-corrected standardized mean difference between sexes with 95% confidence intervals. Hedges’ g was calculated as Male–Female. Positive values indicate higher scores among males. Given the exploratory design and small sample size (n = 3 per group), emphasis is placed on effect magnitude and direction rather than statistical inference.

**Table 4:** Dynamic balance performance in Elite Sunfish Sailors (n = 6).

Variable	Anterior			Posteromedial			Posterolateral			Composite		
	Male	Female	g [95% CI]	Male	Female	g [95% CI]	Male	Female	g [95% CI]	Male	Female	g [95% CI]
DS	77.57 ± 5.29	77.65 ± 6.63	−0.01 [−4.07–1.93]	131.09 ± 2.61	130.58 ± 5.12	0.01 [−2.24–3.69]	128.79 ± 11.77	125.61 ± 3.12	0.29 [−3.24–6.06]	111.28 ± 1.15	112.48 ± 4.64	0.28 [−3.02–5.82]
NDS	73.98 ± 5.47	78.48 ± 5.18	−0.67 [−15.53–0.42]	129.25 ± 12.01	128.08 ± 2.80	0.11 [−3.76–5.54]	115.67 ± 3.78	103.34 ± 4.04	2.52 [2.15–12.41]	123.12 ± 17.61	138.27 ± 2.51	−0.96 [−15.50–1.31]

DS: dominant side; NDS: non-dominant side; CI: confidence interval. Values are presented as mean ± standard deviation. Hedges’ g represents the bias-corrected standardized mean difference between sexes with 95% confidence intervals. Hedges’ g was calculated as Male–Female. positive values indicate higher scores among males. Due to the exploratory design and small sample size (n = 3 per group), findings are interpreted descriptively, emphasizing magnitude and direction of effects. Y Balance Test distances are expressed as a percentage of limb length.

## Data Availability

The datasets used and/or analyzed during the current study are available from the corresponding author upon reasonable request.

## Supplementary Materials

The following supporting information can be downloaded at https://www.mdpi.com/article/10.3390/sports14050182/s1, Table S1: Neuromuscular performance assessment of male and female Elite Sunfish Sailors (n = 6).
