# Supplementary material for: Neuromuscular Performance Characteristics of Elite Colombian Sunfish Sailors: A Pilot Study
Source: Sports (Basel). 2026 May 5;14(5):182. doi: 10.3390/sports14050182 (PMC13211025; doi:10.3390/sports14050182)
Supplement: Supplementary file 1 [file sports-14-00182-s001.zip › sports-4267807-supplementary.pdf]

Supplementary Materials

Table S1. Neuromuscular performance assessment of male and female Elite Sunfish Sailors (n = 6).

Variables	Male (mean ± SD)	Female (mean ± SD)	g [95% CI]
D-Handgrip Strength (Kg-f)	50.73 ± 1.87	31.42 ± 4.09	4.85 [3.96 – 24.77]
ND- Handgrip Strength (Kg-f)	47.02 ± 7.87	29.63 ± 3.60	2.27 [1.77 – 8.60]
Trunk Flexion (min)	5.34 ± 5.23	6.17 ± 3.40	−0.15 [−2.43 – 1.68]
R- Trunk lateral flexion (min)	1.75 ± 0.70	1.95 ± 0.65	−0.24 [−2.27 – 1.42]
L- Trunk lateral flexion (min)	2.03 ± 0.52	2.03 ± 0.68	0.00 [−6.02 – 1.92]
Trunk extension (min)	2.55 ± 0.06	2.54 ± 0.61	0.00 [−10.80 – 7.09]
D- Quadriceps isometric strength (kg)	734.95 ± 165.24	460.10 ±182.17	1.26 [0.43 – 14.87]
ND- Quadriceps isometric strength (kg)	706.06 ± 163.63	429.77 ± 166.64	1.33 [0.67 – 4.49]
D-MVIC Quadriceps (N*m)	237.81 ± 40.77	130.81 ± 53.23	1.81[1.21 – 9.26]
D-MVIC Quadriceps (N*m)	229.05 ±45.22	122.21 ± 48.98	1.81 [1.34 – 5.42]
D- MVIC Quadriceps ($N*m*kg^{-1}$)	3.28 ± 0.68	2.10 ± 0.69	1.36 [−0.46 – 5.14]
ND- MVIC Quadriceps ($N*m*kg^{-1}$)	3.17 ± 0.73	1.97 ± 0.65	1.37 [0.60 – 4.78]
1-RM (kg)	111.93 ± 28.9	71.79 ± 10.95	1.43 [0.70 – 6.92]
CMJ- Jump height (cm)	43.41 ± 5.20	28.56 ± 2.75	2.85 [2.56 – 17.76]
CMJ-Maximun force ($Kg* kg^{-1}$)	2.53 ± 0.23	2.36 ± 0.15	0.68 [−1.30 – 3.21]
CMJ- Peak Power ($W* kg^{-1}$)	55.36 ± 7.02	42.37 ± 4.10	1.80 [1.16 – 6.72]
D-RFD $_{0-50}$ ($N*ms^{-1}$)	1821.23 ± 823.73	1186.17 ± 316.08	0.81 [−0.34 – 5.77]
D-RFD $^{0-150}$ ($N*ms^{-1}$)	1166.83 ± 201.76	776.73 ± 227.81	1.45 [0.51 – 10.52]
D-RFD $_{0-250}$ ($N*ms^{-1}$)	848.13 ± 209.71	548.87 ± 273.82	0.98 [−0.03 – 3.51]
ND-RFD $_{0-50}$ ($N*ms^{-1}$)	1082.03 ± 76.83	1631.51 ± 570.34	1.08 [17.78 – −0.95]
ND-RFD $^{0-150}$ ($N*ms^{-1}$)	911.83 ± 82.79	956.43 ± 383.51	−0.13 [−5.81 – 4.51]
ND-RFD $^{0-250}$ ($N*ms^{-1}$)	769.02 ± 90.38	579.53 ± 285.63	0.71 [−0.52 – 6.48]

CI: confidence interval. Values are presented as mean ± standard deviation. Hedges' g represents the bias-corrected standardized mean difference between sexes. Hedges' g was calculated as Male – Female. Positive values indicate higher scores in males. Given the exploratory design and small sample size (n = 3 per group), emphasis is placed on effect magnitude and direction rather than statistical inference.
